# Association of TLR4 Polymorphisms with Increased Susceptibility to Recurrent Vulvovaginal Candidiasis in Greek Women

**DOI:** 10.3390/microorganisms14030727

**Published:** 2026-03-23

**Authors:** Maria Mavrouli, Chrysoula Verra, Athanasios Tsakris, John Routsias

**Affiliations:** 1 Department of Microbiology, Medical School, National and Kapodistrian University of Athens, 11527 Athens, Greece; atsakris@gmail.com (A.T.); jroutsias@med.uoa.gr (J.R.); 2 Department of Microbiology, General Maternity Hospital “Elena Venizelou”, 11521 Athens, Greece; chrysoula.verra@gmail.com

**Keywords:** RVVC, TLR2, TLR4, single nucleotide polymorphism, vulvovaginal candidiasis, Taqman SNP assay

## Abstract

Recurrent vulvovaginal candidiasis (RVVC) affects 5–8% of women of reproductive age. Host genetic factors, particularly single nucleotide polymorphisms (SNPs) in Toll-like receptors (TLRs), may influence RVVC susceptibility by impairing vaginal mucosal antifungal immunity. The aim of this study was to assess the effect of SNPs in genes encoding TLRs on RVVC susceptibility. Τhe distribution of TLR2 Arg753Gln and TLR4 Asp299Gly/Thr399Ile polymorphisms in Greek women, including RVVC (*n* = 63), first-episode VVC (*n* = 37), *Gardnerella vaginalis* vaginitis (GV, *n* = 36) patients, and healthy controls (*n* = 61), was investigated using TaqMan SNP genotyping. Genotype and allele frequencies were analyzed under allelic and dominant models, with odds ratios (ORs), 95% confidence intervals (CIs), and linkage disequilibrium assessed. TLR4 Asp299Gly and Thr399Ile heterozygotes were significantly more frequent in RVVC patients compared with controls and affected RVVC susceptibility (OR: 5.57, 95% CI: 1.17–26.56, *p*: 0.0172; OR: 4.92, 95% CI: 1.02–23.78, *p*: 0.0306, respectively). No associations were observed for TLR2 Arg753Gln or for any SNP with GV or first-episode VVC. TLR4 variants co-segregated, indicating a haplotype effect. TLR4 haplotypes, rather than TLR2 polymorphism, confer increased RVVC susceptibility, supporting a genetically distinct, mucosal immunity-driven pathogenesis. Larger, ethnically diverse studies with functional assays are warranted to validate these findings and guide personalized prevention and treatment strategies.

## 1. Introduction

Vulvovaginal candidiasis (VVC) is a fungal infection affecting the vaginal and vulvar tissues of the female genital system, primarily caused by the opportunistic yeast *Candida albicans*, which accounts for the majority of reported cases [1,2]. Most women of reproductive age experience at least one episode of VVC in their entire life, whereas approximately 5–8% present with recurrent vulvovaginal candidiasis (RVVC), characterized by the occurrence of three or more symptomatic VVC episodes within a single year [3]. While most women indicate suffering from RVVC for 1 to 2 years, some endure persistent infections for up to 5 years or even for decades [3]. The burden of RVVC extends beyond physical symptoms, as it negatively affects quality of life, leading to high levels of anxiety and depression, social and sexual dysfunction, and significant economic costs, including increased healthcare expenses and loss of productivity [4,5,6,7].

Pathogen exposure alone cannot account for the high recurrence incidence of RVVC, indicating that host-related factors are crucial for both disease susceptibility and persistence [8,9]. Major risk factors of VVC include increased estrogen levels found in pregnant women, uncontrolled diabetes mellitus and treatment with broad-spectrum antibiotics, immunosuppressive drugs and glucocorticosteroids [10,11,12,13,14]. Most RVVC patients, however, are healthy women with no identifiable risk factors that could predispose them to or trigger a new episode [8,15]. Furthermore, not all women who are diagnosed with VVC will eventually develop RVVC, indicating that host susceptibility may be significantly affected by genetic variation [8,16,17,18].

The vaginal mucosa is protected from infections by the innate immune system that provides the first line of defense since it recognizes and responds to microbial pathogens through pattern-recognition receptors (PRRs) [19,20]. Among these, Toll-like receptors (TLRs) detect conserved microbial structures known as pathogen-associated molecular patterns (PAMPs) and activate intracellular signaling cascades through the MyD88-dependent and the TRIF/TRAM-dependent pathways [21,22,23,24,25,26]. These pathways subsequently activate downstream signal transduction cascades, including NF-κB, MAPK, and IRF pathways that stimulate production of inflammatory cytokines and antimicrobial peptides, recruitment of immune cells, and induction of adaptive immune responses, facilitating phagocytosis and the subsequent clearance of the pathogen [21,22,23,24,25,26].

During vulvovaginal infection, TLR2 and TLR4 recognize components of the *Candida* cell wall, all vital for cell shape, integrity, and host interactions, including phospholipomannan, β-glucans, and mannan, leading to the activation of inflammatory pathways and cytokine production, and the eradication of the fungus [8,27,28,29].

Single-nucleotide polymorphisms (SNPs) in the genes encoding TLR2 and TLR4 contribute to variations in immune responses and influence susceptibility to a variety of infectious and inflammatory diseases [18,30,31]. The most extensively studied TLR SNPs are the two nonsynonymous, frequently co-segregating polymorphisms, Asp299Gly and Thr399Ile, in the *TLR4* gene that have been shown to alter the extracellular region of the receptors, affecting their recognition and ligation capabilities. Additionally, the SNP Arg753Glu in the *TLR2* gene has been found to interfere with receptor signaling and decrease cytokine production, compromising host defense mechanisms [18,30,32,33,34].

A growing body of evidence indicates that SNPs in *TLR* genes can affect the risk of VVC and RVVC by modifying the intensity and efficiency of the immune response to *Candida* and other vaginal pathogens, allowing *Candida* to persist, evade clearance, and establish recurrent infection [18,35,36].

The present study aimed to examine the distribution of the TLR2 Arg753Gln (rs5743708), the TLR4 Asp299Gly (rs4986790) and TLR4 Thr399Ile (rs4986791) SNPs in Greek women of reproductive age diagnosed with RVVC, first-episode VVC, *Gardnerella vaginalis*–associated vaginitis (GV), and in healthy controls, using TaqMan SNP genotyping. In addition, comprehensive statistical analysis was conducted to assess whether these variants are associated with an increased genetic risk of vaginal infections, particularly RVVC.

## 2. Materials and Methods

### 2.1. Ethics Statement

The inclusion of patients and controls in this study, as well as the research protocol, were approved by the Committee on Bioethics and Deontology of the Medical School of the National and Kapodistrian University of Athens (Approval Code: 423, Approval Date: 21 January 2021) and the Scientific Council of the General Hospital “Elena Venizelou” where the clinical samples were collected (Approval Code: 29052, Approval Date: 9 December 2020). Participants provided written informed consent, and the study was conducted in accordance with the Declaration of Helsinki.

### 2.2. Clinical Samples

Vaginal swabs were collected from 197 women over 18 years of age, during routine gynecological examination as part of preventive gynecological screening or clinical evaluation for recurrent episodes of fungal vaginitis at the General Maternity Hospital “Elena Venizelou” in Athens, Greece. Patients were considered to have recurrent infections if their medical records showed three or more symptomatic episodes in a year, all of which were microbiologically verified and caused by *C. albicans*. Sixty-three women with RVVC, 37 with first-onset VVC and 36 with non-specific vaginitis caused by *Gardnerella vaginalis* (GV) were examined for the presence of the TLR2 Arg753Gln (rs5743708) polymorphism and the TLR4 Asp299Gly (rs4986790) and Thr399Ile (rs4986791) polymorphisms. The control group consisted of sixty-one women who had no current gynecological complaints, no history of vaginal infections and were negative for pathogen cultures. Every participant was of reproductive age and Greek nationality. Exclusion criteria for selection included pregnancy, diabetes mellitus, use of immunosuppressive drugs and long-term treatment with broad-spectrum antibiotics.

### 2.3. Direct Microscopy and Culture of Vaginal Specimens

Vaginal specimens were collected by gynecologists using two sterile cotton swabs from the posterior vaginal fornix, one for direct microscopy examination and the other for microbiological culture. Microscopic examination was performed using a saline wet mount preparation to detect fungal elements. The presence of budding yeast cells was considered suggestive of *Candida* spp.

All media used for bacterial and fungal culture were commercially purchased and used according to the manufacturer’s instructions. For bacterial culture, the samples were inoculated onto Columbia agar with 5% sheep blood, chocolate agar with vitox and MacConkey agar plates (Oxoid, Thermo Fisher Scientific, Wesel, Germany) and incubated at 37 °C for 24 h under appropriate atmospheric conditions. These media were used to allow detection and differentiation of a broad range of bacterial species commonly associated with vaginal infections.

For fungal isolation, samples were cultured on CHROMID^®^ Candida agar (BioMérieux Inc., Durham, NC, USA) and incubated at 37 °C for 24 h to promote the optimal growth of clinically relevant yeast species, particularly *C. albicans* which grows efficiently at human body temperature. Following inoculation of CHROMID^®^ Candida agar, the swab was immersed in 1–2 mL thioglycolate enrichment broth (Oxoid, Thermo Fisher Scientific, Wesel, Germany) and was incubated at room temperature (approximately 22–25 °C) for 24 h to enhance the recovery of microorganisms in low numbers. Following enrichment, subculture on CHROMID^®^ Candida agar plates was performed selectively for samples considered suspicious for fungal presence at 37 °C for 24 h. This selection was based either on the patient’s clinical history suggesting possible fungal infection or on the observation of occasional fungal elements during direct microscopic examination. *Candida* species were identified based on colony morphology and characteristic color development on CHROMID^®^ Candida agar, which allows differentiation of *C. albicans* from other *Candida* species.

### 2.4. Genomic DNA Isolation

The isolation of genomic DNA from nucleated epithelial cells acquired via vaginal swabs was performed with the NucleoSpin^®^ Tissue kit (Macherey-Nagel, Düren, Germany) according to the manufacturer’s instructions. After microscopic examination, each swab was immersed in a sterile tube containing 2 mL of phosphate-buffered saline (PBS) and incubated for approximately 2–3 h at room temperature (approximately 22–25 °C) to facilitate the release of epithelial cells from the swab fibers into the suspension. The tubes were then vortexed briefly to enhance cell detachment. After centrifuging the suspension for 5 min at 13,000× *g*, the supernatant was carefully discarded, and the pellet, containing epithelial cells, was subjected to genomic DNA extraction using the standard protocol for human or animal tissue and cultured cells provided in the NucleoSpin^®^ Tissue kit. In brief, the pellet was lysed using the provided lysis buffer in the presence of 25 μL proteinase K (56 °C, 1–3 h), allowing digestion of cellular proteins and release of genomic DNA. Following lysis, ethanol (96–100%) was added to promote DNA binding, and the lysate was transferred to a silica membrane spin column, where genomic DNA selectively binds under chaotropic salt conditions. The column was subsequently subjected to sequential washing steps to remove proteins, salts, and other contaminants. Finally, purified genomic DNA was eluted in 100 μL nuclease-free elution buffer and stored at −20 °C until further molecular analysis.

### 2.5. Purity Control and Quantification of Nucleic Acids

Genomic DNA samples were evaluated for concentration, purity, and suitability for amplification prior to real-time qPCR analysis. By measuring absorbance at 260 nm and calculating the A260/A280 ratio with UV spectrophotometry (NanoPhotometer^TM^ P-Class, Implen GmbH, Munich, Germany), the quantity and purity of DNA were determined. Measurements were performed using 2 μL of undiluted DNA sample, and the spectrophotometer was blanked with the same solution used for DNA elution to ensure accurate baseline correction. All samples were subsequently normalized to a final concentration of 10 ng genomic DNA per reaction using nuclease-free water to ensure uniform amplification conditions across samples and comparability between reactions. To guarantee accurate and repeatable amplification findings, only DNA samples with an A260/A280 ratio between 1.8 and 2.0 and sufficient DNA concentration were included in this qPCR analysis. Additionally, amplification of the human housekeeping *ACTB* gene (β-actin) was performed by real-time PCR as an internal control to verify the presence and quality of genomic DNA prior to SNP genotyping. Samples failing to amplify the internal control were excluded from further analysis.

### 2.6. TLR2 and TLR4 SNP Genotyping

The genomic DNA used for genotyping was isolated from nucleated epithelial cells collected from vaginal swabs. Although vaginal specimens may contain microbial DNA, the TaqMan SNP genotyping assays used in this study are highly specific for human genomic sequences, particularly human SNP loci, and they do not amplify fungal DNA. Genotyping of TLR2 Arg753Gln (rs5743708), TLR4 Asp299Gly (rs4986790) and TLR4 Thr399Ile (rs4986791) polymorphisms was performed using pre-designed Taqman SNP genotyping assays (Applied Biosystems, Life Technologies, Carlsbad, CA, USA), which include sequence-specific primers and two allele-specific fluorescently labeled Minor Groove Binder (MGB) probes for discrimination between wild-type and variant (mutated-type) alleles (Table 1). Primer and probe sequences are proprietary to the manufacturer. The genotype of the TLR2 Arg753Gln (rs5743708) polymorphism was assessed by applying the TaqMan SNP assay C_27860663_10 (Applied Biosystems, Life Technologies, Carlsbad, CA, USA). The genotyping for the TLR4 polymorphisms Asp299Gly (rs4986790) and Thr399Ile (rs4986791) was performed with the TaqMan SNP assay C_11722238_20 and C_11722237_20, respectively (Applied Biosystems, Life Technologies, Carlsbad, CA, USA). PCR reactions were carried out in a final volume of 25 μL containing 12.5 μL iQ Supermix (Bio-Rad Laboratories, Inc., Hercules, CA, USA), 2.5 μL TaqMan SNP genotyping assay mix (Applied Biosystems, Foster City, CA, USA), 10 ng genomic DNA and nuclease-free water to reach the final volume. The TaqMan qPCR assays were performed on the AriaDx Real-time PCR system (Agilent Technologies, Santa Clara, CA, USA). The cycling protocol consisted of an initial enzyme activation step at 95 °C for 10 min, followed by 40 cycles of denaturation at 95 °C for 15 s and combined annealing/extension at 60 °C for 1 min. Allelic discrimination analysis was conducted using the Agilent Aria V2.0 software by evaluating the fluorescence signals of the reporter dyes, allowing classification of samples into homozygous or heterozygous genotypes. A random subset of samples was analyzed in duplicate to ensure reproducibility of the results. Each qPCR run included no-template controls (NTCs) containing nuclease-free water to detect potential contamination.

### 2.7. Statistical Analysis

Genotype and allele frequencies were compared between patients and healthy controls using the chi-square test or Fisher’s exact test, as appropriate. Due to small expected cell counts and the absence of minor allele homozygotes, Fisher’s exact test was considered the primary method for assessing statistical significance. The association between SNPs and disease susceptibility was evaluated by calculating odds ratios (ORs) with corresponding 95% confidence intervals (95% CIs) under dominant and allelic genetic models. Chi-square test or Fisher’s exact test and calculation of ORs with corresponding 95% CIs were performed using GraphPad Prism v9.0 (Boston, MA, USA). A statistically significant association was considered present when Fisher’s exact test yielded a *p*-value < 0.05 and the corresponding 95% CIs of the odds ratio did not include 1.0. Hardy–Weinberg equilibrium (HWE) was assessed in both cases and controls using the OEGE online Hardy–Weinberg equilibrium calculator. Linkage disequilibrium (LD) between polymorphisms was evaluated by calculating D and r^2^ coefficients using standard mathematical formulas based on observed allele and haplotype frequencies. The calculations were implemented in Microsoft Excel (Microsoft Corp., Redmond, WA, USA; version 365).

## 3. Results

The genotype distributions of TLR2 Arg753Gln, TLR4 Asp299Gly and TLR4 Thr399Ile among patients and healthy individuals are presented in Table 2. No homozygotes for the minor allele were detected for any of the examined SNPs in either patients or controls, indicating a low minor allele (mut/mut) frequency in the studied population. Most individuals in all groups are wild-type (wt/wt), which strengthens the above observation, thus the differences between groups concern exclusively heterozygous individuals (wt/mut).

For TLR2 Arg753Gln, the frequency of the heterozygous genotype was low and comparable across all groups (Controls: 3.3%; GV: 2.8%; VVC: 8.1%; RVVC: 4.8%), suggesting no apparent difference between patients and healthy individuals. In contrast, TLR4 Asp299Gly exhibited a higher frequency of heterozygotes in the RVVC group (15.9%) compared to the control group (3.3%), while the frequencies in GV (5.6%) and VVC groups (5.4%) were comparable to that observed in controls. A similar distribution pattern was observed for TLR4 Thr399Ile, with an increased heterozygous frequency in RVVC (14.3%) compared to controls (3.3%), while GV (5.6%) and VVC (5.4%) showed frequencies similar to the control group. The distribution patterns suggest a potential association of TLR4 Asp299Gly and TLR4 Thr399Ile with RVVC, whereas no clear association is evident for TLR2 Arg753Gln or any of the three SNPs with GV and VVC.

To further investigate the association between the studied polymorphisms and disease susceptibility, odds ratios (ORs) with 95% confidence intervals (95% CIs) were calculated under both allelic and dominant models. Statistical significance was additionally assessed using Fisher’s exact test, and the corresponding *p*-values were determined for each model. In the dominant genetic model, heterozygous and homozygous variant genotypes were grouped together and compared with the wild-type genotype. Since no individuals were homozygous for the minor allele, the allelic and dominant models yielded nearly identical results, as all minor allele carriers were heterozygotes.

No significant association was observed between TLR2 Arg753Gln and the occurrence of GV (*p* = 0.6909), VVC (*p* = 0.3627) or RVVC (*p* = 0.5154) compared to healthy individuals. Similarly, TLR4 Asp299Gly was not significantly associated with susceptibility to GV (*p* = 0.4754) or VVC (*p* = 0.4869). In contrast, a statistically significant association was identified between TLR4 Asp299Gly and susceptibility to RVVC (*p* = 0.0172). Regarding TLR4 Thr399Ile, no significant association was detected with GV (*p* = 0.4754) or VVC (*p* = 0.4869). However, a statistically significant association was observed between TLR4 Thr399Ile and RVVC susceptibility (*p* = 0.0306).

To determine whether the observed associations resulted in an increased risk of disease occurrence, effect size estimates were obtained through calculation of ORs and 95% CIs. Statistical significance was defined as a 95% CI that did not include 1.0. An OR greater than 1.0 indicates an increased disease risk associated with the genetic variant examined, whereas an OR close to 1.0 suggests no meaningful association.

For TLR2 Arg753Gln, the 95% CI included 1.0 across all disease groups, indicating no significant association with increased risk for GV, VVC, or RVVC (Table 3). For TLR4 Asp299Gly, no significant association was observed with GV or VVC, as the 95% CI included 1.0. In contrast, in RVVC group, the 95% CI excluded 1.0, indicating a statistically significant association and an increased risk of disease occurrence. Individuals carrying the heterozygous genotype exhibited an approximately sixfold increased odds of RVVC (OR: 5.57; 95% CI: 1.17–26.56) compared with wild-type individuals.

Similarly, for TLR4 Thr399Ile, the 95% CI included 1.0 for GV and VVC, indicating no statistically significant association. However, in RVVC group, the 95% CI excluded 1.0, demonstrating a statistically significant association with increased risk. Carriers of the heterozygous genotype demonstrated approximately fivefold higher odds of developing RVVC (OR: 4.92; 95% CI: 1.02–23.78).

Genotype frequencies of all three polymorphisms were in Hardy–Weinberg equilibrium in both patient and control groups (*p* > 0.05). In the RVVC group, it was found that among patients who were heterozygous for TLR4 Asp299Gly, 90% were also heterozygous for TLR4 Thr399Ile (Table 2). A significant linkage disequilibrium (D = 0.120, r^2^ = 0.88) between TLR4 Asp299Gly and TLR4 Thr399Ile was observed, suggesting co-segregation of these variants.

## 4. Discussion

Host genetic variability, particularly single nucleotide polymorphisms (SNPs), significantly influences innate immune responses and susceptibility to fungal diseases [37]. Variants in genes regulating cytokine production, intracellular signaling, and cell adhesion have been associated with RVVC predisposition [38]. Polymorphisms affecting pattern recognition receptors (PRRs), including TLRs and mannose-binding lectin (MBL), have been identified in women with RVVC and are linked to increased vulnerability to VVC [17,36,39,40,41,42,43,44]. Specifically, TLR2 and TLR4 polymorphisms have been associated with altered immune responses to *Candida* species, supporting a role for genetic predisposition in RVVC pathogenesis [33,35,36,41].

The contribution of TLR2 and TLR4 polymorphisms to susceptibility to fungal infections has been extensively investigated, although findings vary depending on the pathogen and clinical presentation. In invasive fungal diseases such as aspergillosis, TLR4 polymorphisms (Asp299Gly and Thr399Ile) have been associated with increased susceptibility to infection [37,45]. A significant association was observed between TLR4 Asp299Gly (allele G) and chronic cavitary pulmonary aspergillosis (OR: 3.46, *p* = 0.003) [37]. Genetic variation in TLR4 significantly increases risk of invasive aspergillosis especially in immunocompromised hosts [45]. Particularly, the Asp299Gly/Thr399Ile haplotype in stem cell donors has been associated with increased susceptibility to invasive aspergillosis in recipients of allogeneic hematopoietic stem cell transplants, although the underlying mechanisms remain unclear [45]. It has been proposed that these variants may alter cytokine production and thereby modulate antifungal immune responses [18,35]. Structural studies indicate that the Asp299Gly substitution induces localized conformational changes on the surface of TLR4, potentially influencing responsiveness to ligands with weak agonistic activity, whereas the Thr399Ile variant appears to a minimal structural and functional impact on TLR4 [18,46].

TLR2 plays a key role in antifungal immunity by recognizing fungal components such as β-glucans and mediating host responses against pathogens like *C. albicans* and *Aspergillus* spp. [28,47,48]. Genetic polymorphisms in TLR2 and related innate immune receptors have been associated with increased susceptibility to invasive fungal infections, particularly in immunocompromised individuals, likely through impaired cytokine responses (e.g., reduced IFN-γ and IL-8) [30,37,49,50,51]. Specific variants, such as R753Q TLR2 polymorphism, have been associated with increased risk of candidemia [52]. The TLR2 Pro631His (rs5743704) polymorphism has been associated with an approximately threefold increased risk of RVVC, further supported by experimental evidence showing that TLR2 deficiency impairs antifungal defense [36,53].

Functional studies have demonstrated that the TLR2 Pro631His polymorphism impairs antifungal cytokine responses, including reduced IL-17 and IFN-γ production, although these findings derive primarily from peripheral immune cells [36]. While TLR2 and TLR4 signaling are clearly active in vaginal epithelial cells and regulate local inflammatory responses during *C. albicans* infection [18,54], the direct impact of TLR2 and TLR4 polymorphisms on epithelial responses in RVVC has not yet been experimentally investigated in the vaginal mucosal context.

Given the crucial role of TLR2 and TLR4 in mediating antifungal and antibacterial immune responses at the vaginal mucosa, this study investigated whether SNPs in genes encoding the innate immune receptors TLR2 and TLR4 contribute to susceptibility to vulvovaginal infections, particularly RVVC, in Greek women. Polymorphisms in genes encoding TLR2 and TLR4 are biologically plausible candidates for modulating host–pathogen interactions on vaginal mucosa considering the established effects of TLR4 Asp299Gly and Thr399Ile variants on ligand recognition and subsequent immunological activation, as well as the impact of the TLR2 Arg753Gln variant on receptor signaling [18].

Three clinically and microbiologically distinct vaginal diseases, including RVVC, first-episode VVC, and nonspecific bacterial vaginosis caused by *Gardnerella vaginalis* (GV), were simultaneously genetically investigated alongside healthy controls. Although previous genetic studies have either focused exclusively on RVVC [17,36,42,55] or analyzed heterogeneous vaginitis phenotypes as a single group [56,57,58,59], the current study clearly distinguishes among these diseases. Our findings demonstrate a statistically significant association between the TLR4 SNPs, Asp299Gly and Thr399Ile, and susceptibility to RVVC (*p* = 0.0172 and *p* = 0.0306, respectively), suggesting that TLR4 genetic variation may play a role in disease pathogenesis. Individuals with the heterozygous Asp299Gly genotype exhibited a 5.57-fold greater risk of RVVC (OR: 5.57; 95% CI: 1.17–26.56), whereas heterozygous Thr399Ile carriers had a 4.92-fold increased risk of developing RVVC (OR: 4.92; 95% CI: 1.02–23.78). It is well known that these SNPs impair innate immune signaling mediated by TLR4 [30,32]. Their strong association with RVVC provides a plausible explanation for the reduced clearance of *Candida* species observed in affected women [36,41,55,60], despite the absence of systemic immunodeficiency [61] and supports the hypothesis that defective innate mucosal immunity underlies chronic or recurrent disease [8,62].

In European populations, TLR4 Asp299Gly and TLR4 Thr399Ile are often inherited together, forming a haplotype associated with altered TLR4 signaling and modified innate immune responses [30,32,63]. In this study, a strong linkage disequilibrium (D = 0.120, r^2^ = 0.88) was observed between these two TLR4 SNPs supporting their cosegregation within the study population. Although formal haplotype frequency estimation was not performed in this study, the high r^2^ value along with the nearly identical distribution of the heterozygous genotypes for the two TLR4 polymorphisms across all study groups strongly suggests that these polymorphisms are frequently co-inherited, which is consistent with previous observations particularly in European (Caucasian) populations [33]. Thus, susceptibility to RVVC may not be attributable to isolated single nucleotide variants, but rather to TLR4 haplotypes that exert a combined effect on TLR4 function and may collectively undermine antifungal innate immune responses at the vaginal mucosal surface. Since all participants were of Greek origin, population stratification is expected to be minimal. However, larger studies including formal haplotype reconstruction would be useful to further clarify the contribution of TLR4 haplotypes to RVVC susceptibility.

In contrast, no association was detected between TLR2 Arg753Gln and RVVC, nor were any of the three studied SNPs associated with GV or VVC. These findings strongly support the concept that RVVC represents a genetically and immunologically distinct clinical entity, rather than a more severe manifestation of sporadic or acute VVC [55,61]. Furthermore, these *TLR4* genetic variants likely influence susceptibility in a disease-specific manner, rather than acting as a broad, universal risk factor for vaginal infection in general. The absence of correlation between the TLR2 Arg753Gln and RVVC, VVC, or GV may indicate that alternative pattern recognition receptors in the vaginal mucosa may sufficiently compensate for impaired TLR2 signaling, despite the recognized role of TLR2 in *Candida* recognition [30]. The cooperative interactions between TLRs and C-type lectin receptors likely maintain the antifungal response when a single signal transduction pathway is disrupted [28,29]. The significant correlation observed between TLR4 variants and RVVC highlights the important role of TLR4-mediated signaling in maintaining antifungal immunity in this anatomical region [55,61]. Furthermore, these findings support the hypothesis that different pathways of the innate immune system do not contribute equally to susceptibility to RVVC and that selective deficiencies may differentially affect disease risk.

Our findings support a host-genetic model of RVVC pathogenesis. The identification of host genetic factors predisposing to RVVC enhances our understanding of disease pathogenesis and may facilitate the development of personalized approaches to prevention, diagnosis, and treatment. Genotypic identification of women carrying TLR4 SNPs could enable early recognition of individuals at increased risk for RVVC, thereby allowing closer monitoring, implementation of targeted preventive measures, or personalized treatment strategies. In the future, therapeutic approaches might include immunomodulators or antifungal therapies tailored to individual genetic profiles aimed at improving TLR4-mediated signaling or compensating for functional deficiencies in genetically susceptible women.

Despite its strengths, the study has certain limitations that should be acknowledged. TLR4 and TLR2 SNPs are relatively uncommon in European populations [32,63], resulting in small numbers of heterozygous individuals, particularly among healthy women and GV or VVC patients. Although the associations with RVVC are statistically significant, the odds ratio estimates are characterized by wide confidence intervals, reflecting limited precision.

In addition, no homozygous mutant genotypes were observed, preventing analysis of gene-dosage effects, recessive inheritance patterns, or whether carrying two mutated alleles would further increase the risk of RVVC. The study also lacked functional immune assays, such as cytokine profiling or TLR expression analysis, which could have provided direct evidence of the biological impact of these SNPs in vaginal tissue. Although TLR4 variants are known to negatively affect immunological signaling, the study could not clearly determine how these mutations affected vaginal mucosa immune responses in the participants. Finally, the study population consisted exclusively of Greek women; therefore, the findings may not be directly generalizable to other ethnic groups, including non-European or non-Mediterranean populations, with different genetic backgrounds. Our findings highlight the need for further studies in different ethnic populations with larger numbers of participants to validate and extend the observed associations.

## 5. Conclusions

This study supports a host-genetic model of RVVC pathogenesis, in which inherited variations in innate immune signaling pathways contribute to susceptibility. TLR4 SNPs, rather than TLR2 polymorphism, are associated with increased risk of RVVC occurrence. RVVC appears to be a genetically and immunologically distinct condition, rather than a more severe form of sporadic or acute infection.

Future studies in larger, ethnically diverse cohorts combined with functional immune analyses are needed to validate these associations and clarify the biological mechanisms underlying RVVC. Understanding the host-genetic factors that predispose to RVVC and early identification of women carrying high-risk TLR4 variants may facilitate personalized prevention, monitoring, and therapeutic strategies, including immunomodulatory approaches to enhance local antifungal immunity.

## Figures and Tables

**Table 1:** Details regarding the TLR2 and TLR4 SNPs examined, according to the manufacturer (Applied Biosystems, Life Technologies, Carlsbad, CA, USA).

	TLR2 Arg753Gln	TLR4 Asp299Gly	TLR4 Thr399Ile
SNP ID	rs5743708	rs4986790	rs4986791
Assay ID	C__27860663_10	C__11722238_20	C__11722237_20
Gene	*RNF175 TLR2*	*TLR4*	*TLR4*
Gene name	ring finger protein 175	toll-like receptor 4	toll-like receptor 4
Chromosome Location	Chr.4: 153705165 on Build GRCh38, third exon	Chr.9: 117713024 on Build GRCh38, third exon	Chr.9: 117713324 on Build GRCh38, third exon
Polymorphism	G/A, transition substitution at position 2258	A/G, transition substitution at position 896	C/T, transition substitution at position 1196
Fluorescent dyes	VIC: mutated type allele, FAM: wild type allele	VIC: wild type allele, FAM: mutated type allele	VIC: wild type allele, FAM: mutated type allele

**Table 2:** Distribution of TLR2 and TLR4 genotypes among controls and patient groups. Values represent number of individuals (percentage within each group). Percentages were calculated based on the total number of individuals in each group. No homozygous mutant genotype was detected for any of the polymorphisms investigated in the study population.

SNPs	Group (*n*)	Wild-Type (wt/wt)	Heterozygous (wt/mut)	Homozygous (mut/mut)
TLR2 Arg753Gln rs5743708 (G → A)		GG	GA	AA
	Controls (61)	59 (96.7%)	2 (3.3%)	0 (0.0%)
	GV (36)	35 (97.2%)	1 (2.8%)	0 (0.0%)
	VVC (37)	34 (91.9%)	3 (8.1%)	0 (0.0%)
	RVVC (63)	60 (95.2%)	3 (4.8%)	0 (0.0%)
TLR4 Asp299Gly rs4986790 (A → G)		AA	AG	GG
	Controls (61)	59 (96.7%)	2 (3.3%)	0 (0.0%)
	GV (36)	34 (94.4%)	2 (5.6%)	0 (0.0%)
	VVC (37)	35 (94.6%)	2 (5.4%)	0 (0.0%)
	RVVC (63)	53 (84.1%)	**10 (15.9%)**	0 (0.0%)
TLR4 Thr399Ile rs4986791 (C → T)		CC	CT	TT
	Controls (61)	59 (96.7%)	2 (3.3%)	0 (0.0%)
	GV (36)	34 (94.4%)	2 (5.6%)	0 (0.0%)
	VVC (37)	35 (94.6%)	2 (5.4%)	0 (0.0%)
	RVVC (63)	54 (85.7%)	**9 (14.3%)**	0 (0.0%)

**Table 3:** Genetic association of SNPs in *TLR2* and *TLR4* genes with GV, VVC or RVVC susceptibility was evaluated by calculating odds ratios (ORs) with corresponding 95% confidence intervals (95% CIs) using healthy controls as the reference group. Odds ratios were calculated using the variant allele (A for TLR2 rs5743708, G for TLR4 rs4986790, and T for TLR4 rs4986791) as the risk allele. Allelic and dominant genetic models were applied. In the dominant model, heterozygous and homozygous variant genotypes were grouped together and compared with the wild-type genotype. *p*-values were calculated using Fisher’s exact test. Statistically significant results (*p* < 0.05) are shown in bold. * *p* < 0.05 was considered statistically significant.

SNPs	Groups	*p*-Value		OR (95% CI)	
		Allelic	Dominant	Allelic	Dominant
TLR2 rs5743708 Arg753Gln (G → A)	GV	0.6900	0.6909	0.85 (0.08–9.49)	0.84 (0.07–9.64)
	VVC	0.2773	0.3627	2.54 (0.41–15.54)	2.60 (0.41–16.36)
	RVVC	0.5152	0.5154	1.46 (0.24–8.91)	1.48 (0.24–9.15)
TLR4 rs4986790 Asp299Gly (A → G)	GV	0.4749	0.4754	1.71 (0.24–12.44)	1.74 (0.23–12.89)
	VVC	0.4863	0.4869	1.67 (0.23–12.09)	1.69 (0.23–12.51)
	RVVC	0.0196	0.0172 *	5.17 (1.11–24.12)	**5.57 (1.17–26.56)**
TLR4 rs4986791 Thr399Ile (C → T)	GV	0.4749	0.4754	1.71 (0.24–12.44)	1.74 (0.23–12.89)
	VVC	0.4863	0.4869	1.67 (0.23–12.09)	1.69 (0.23–12.51)
	RVVC	0.0337	0.0306 *	4.62 (0.98–21.82)	**4.92 (1.02–23.78)**

## Data Availability

The original contributions presented in this study are included in the article. Further inquiries can be directed to the corresponding author.
